# Conducting a pilot randomized controlled trial of community-based mindfulness-based stress reduction versus usual care for moderate-to-severe migraine: protocol for the Mindfulness and Migraine Study (M&M)

**DOI:** 10.1186/s13063-019-3355-y

**Published:** 2019-05-06

**Authors:** Alice Pressman, Heather Law, Robert Stahl, Alex Scott, Alice Jacobson, Lisa Dean, Sylvia Sudat, Angelica Obillo, Andrew Avins

**Affiliations:** 1 Sutter Health Research Development and Dissemination, 2121 North California Blvd, Walnut Creek, CA 94596 USA; 2 Insight Santa Cruz a Buddhist Meditation Community, 119 Marnell Ave, Santa Cruz, CA 95062 USA; 3 Palo Alto Medical Foundation Research Institute, 795 El Camino Real, Palo Alto, CA 94301 USA; 4 Kaiser Permanente Northern California Division of Research, 2000 Broadway, Oakland, CA 94612 USA

**Keywords:** Migraine, Headache, Pain, Mindfulness, Mindfulness-based stress reduction (MBSR), Pilot, Feasibility, Trial protocol, Behavioral intervention, Pragmatic, RCT

## Abstract

**Background:**

Migraine is one of the most common neurological disorders in clinical practice and is a substantial cause of disability worldwide. Current approaches to therapy are primarily based on medication but are often limited by inadequate effectiveness and common side effects. Newer, more effective medications are expensive. Mindfulness-based stress reduction (MBSR), an 8-week classroom-based meditation intervention, is inexpensive, has no known side effects, and has demonstrated clinically meaningful effectiveness for several chronic-pain syndromes. In addition, MBSR has shown promising results for migraine therapy in a few small case studies and pilot studies. We present here the protocol for a two-arm randomized controlled pilot trial of MBSR for moderate-to-severe episodic migraine, which, if successful, will form the basis for a fully powered clinical trial.

**Methods/design:**

This study, set in Northern California, is a two-arm parallel-comparison single-blinded randomized controlled pilot trial with the goal of recruiting approximately 60 participants with moderate-to-severe episodic migraine. The feasibility outcomes include ability and time required to recruit, adherence to the MBSR treatment, and ability to measure outcomes using 31-day headache diaries and patient-reported questionnaire data. The active treatment arm consists of an 8-week community-based MBSR class plus usual care, and the wait-list control group is usual care. Recruitment is underway and expected to be complete by the end of 2018.

**Discussion:**

To our knowledge, this is the first pragmatic trial in the U.S. of MBSR for migraine using community-based classes, and if it proves viable, we plan to conduct a fully powered trial to determine the effectiveness of the intervention for reducing headache days for moderate-to-severe episodic migraineurs.

**Trial registration:**

Clinicaltrials.gov, NCT02824250. Registered on 6 July 2016.

**Electronic supplementary material:**

The online version of this article (10.1186/s13063-019-3355-y) contains supplementary material, which is available to authorized users.

## Background

Migraine, one of the most common neurological disorders in the U.S., is ranked among the top 20 causes of disability worldwide [1–3]. Migraine is currently one of the leading causes of disease burden for women aged 15–44 years and affects an estimated 11% of the adult population globally, with a strong female predominance [1–4]. The most common approach to therapy is generally focused on pharmaceutical management, which is often limited by insufficient efficacy and frequent side effects. The newest class of migraine-preventive drugs, the calcitonin gene-regulating peptides, while promising, are very expensive and not consistently beneficial for a substantial proportion of the migraine-affected population [5]. Because stress is a common precipitant of migraine episodes, it is rational that a stress-reducing intervention may favorably affect the course of migraine headache [6, 7].

Mindfulness-based stress reduction (MBSR), created in 1979 by Jon Kabat-Zinn at the University of Massachusetts Medical Center, is an 8-week classroom-based intervention that combines mindfulness meditation, yoga, stress psychology and physiology, neuroscience, experiential education, and a group process. Several studies have been published regarding the effects of MBSR on a variety of medical conditions such as chronic pain and mental health [8, 9]. MBSR is inexpensive, can be delivered in a group format, and has no known adverse effects. MBSR has appeared promising in two small case studies and three pilot trials as an effective treatment for moderate-to-severe episodic migraine [10–14]. In particular, one pilot trial of MBSR for patients with episodic migraine found promising outcomes related to the intervention, providing important impetus for future research [11]. Thus, more rigorous and large-scale clinical trials are needed to test the effectiveness of mind-body interventions such as yoga and meditation in improving not only migraine-related pain but mental health outcomes and quality of life for migraineurs. If MBSR is found to be an effective adjunctive therapy for migraine compared to usual care, providers will have an additional safe and inexpensive preventive-treatment option to offer their patients.

We present here the protocol for a two-arm randomized controlled pilot trial of MBSR for patients with moderate-to-severe episodic migraine headaches. The trial is currently in progress. Should this research prove that it is possible to recruit to a trial of community-based MBSR classes and that the treatment is acceptable to participants, the experience gained from this trial will be used to design and implement a fully powered phase III trial with the objective of testing a low-risk intervention to assist patients who suffer from this often debilitating disorder. As this study contains all the elements of a fully powered trial, but with a single site and smaller sample size, it can be classified as a pilot study [15].

This randomized controlled pilot trial (the Mindfulness and Migraine or M&M study) is designed to assess the achievability and acceptability of an 8-week community-based MBSR intervention for patients with moderate-to-severe episodic migraine. The feasibility outcomes include ability and time required to recruit, adherence to the MBSR treatment, and ability to measure outcomes using 31-day headache diaries and patient-reported questionnaire data. Although we are not measuring efficacy, we will collect clinical outcome data to show the feasibility of our data collection methods. The primary clinical outcome is the change from baseline in headache frequency at 4 months derived from headache diaries. Secondary outcomes include change in headache frequency at 8 months post-randomization and other headache-related measures including headache pain, disability, quality of life, and mental health.

## Methods/design

**Fig. 1:**
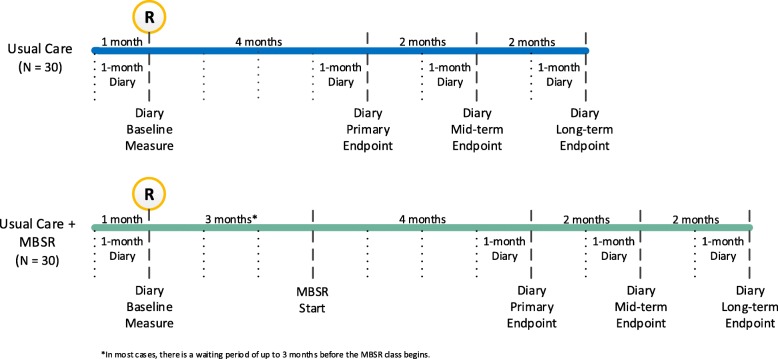
Study flow diagram. MBSR Mindfulness-based stress reduction

This study is a single-center, parallel, randomized, partially blinded, controlled pilot study (Fig. 1), designed to assess the feasibility of conducting a fully powered effectiveness randomized controlled trial.

### Setting and participants

#### Clinical sites

All in-person study recruitment and randomization activities take place at the Palo Alto Medical Foundation Research Institute in Northern California. Participants are recruited from three sources: (1) the Palo Alto Medical Foundation, which is a Sutter Health affiliate; (2) Kaiser Permanente Northern California Redwood City Medical Center; and (3) the greater Palo Alto community. Sutter Health is a large not-for-profit integrated health system in Northern California with five health foundations and 24 hospitals serving more than 3 million patients annually. Palo Alto Medical Foundation, the largest of the five medical foundations, is in Palo Alto, California, and serves approximately 1 million patients annually. Kaiser Permanente Northern California is a large integrated health plan with 4.1 million members. Both Sutter and Kaiser use EpicCare® for their electronic health record (EHR) system. Although most patients are initially identified from the EHR in both systems, the workflow for patient recruitment is different for the two entities due to different regulatory requirements (see details below).

#### Eligibility criteria

Participants are adults with moderately severe migraine headaches who meet the eligibility criteria detailed below. A log of potential participants who decline to enter the study or who are deemed ineligible is kept to judge the representativeness of those who consent to be randomized, consistent with CONSORT guidelines [16].

#### Inclusion criteria

1. Age at least 18 years old
2. Moderate-to-severe migraine headaches, defined as baseline headache frequency of 4–14 headache days per month (over a period of 31 days as determined by the headache diary completed as part of the run-in period)

#### Exclusion criteria

1. Has practiced MBSR or another meditation technique in the past 6 months (daily prayer is not considered a regular meditation practice)
2. Chronic migraine (i.e., > 14 headache days per month).
3. Cognitively or emotionally impaired
4. Pregnant
5. Inability to speak or write in English
6. Started or changed migraine medication (abortive or prophylactic) in the month prior to randomization
7. Incomplete run-in headache diary
8. Insufficient availability to attend an approved community-based MBSR class

#### Recruitment

Multiple recruitment strategies were employed for two reasons. First, we recruited from two different health-care systems with different regulatory requirements to maximize the generalizability of our recruitment strategies. Second, different recruitment strategies were used to give a better understanding of the advantages and challenges of the optimal recruiting techniques for this population in preparation for a larger trial.

#### Recruitment at the Sutter Health site

The Sutter institutional review board (IRB) requires active permission from providers for the recruitment of patients into this study. Potentially eligible patients are identified using an EHR-based electronic migraine probability algorithm previously developed and validated by our group [17]. The criteria for active recruitment include a score > 10 from the migraine probability algorithm and an encounter with the site for migraine in the previous 12 months. Providers are given invitation letters for each patient identified and asked to sign those whom they approve. Study personnel then retrieve and mail the signed letters. Each invitation letter contains a return postcard and gives a toll-free number. If patients return the postcard asking to be contacted or they leave a message on the study phone line, the study coordinator calls them for a preliminary screening.

#### Recruitment at the Kaiser Permanente site

Patients with a history of migraine headache are identified from the EHR. The charts of a random sample of these patients are reviewed by the site’s principal investigator to identify those who have clear evidence of migraine and no EHR-identifiable exclusion criteria. The primary-care physicians for these patients are then contacted and, if there are no objections, each patient is mailed a recruitment letter with an opt-out postcard. Those patients who contact the Kaiser study center are called immediately. Those who do not contact the Kaiser study center and have not returned an opt-out postcard are contacted by phone 3 weeks after the letter is mailed. If a patient passes the telephone screen and expresses interest in participating, their contact information is given to the Sutter Health project coordinator, who contacts the patient and arranges an in-person assessment.

#### Community-based recruitment

Several methods are used for community recruitment, including posting flyers at local coffee shops, train stations, and grocery stores. The study is also posted on clinicaltrials.gov (NCT02824250).

### Randomization

Prior to the start of the study, a balanced, blocked randomization list was generated using Stata v14.0 using variable block sizes of 2, 4, and 6 [18]. From the list, a research assistant not affiliated with the study translated the allocation numbers into groups and placed each allocation into an opaque envelope, labeled with a sequential study identifier. The envelopes were then sealed and transferred to the study site.

When each potential participant returns to the clinic with their completed run-in diary (see below), the study staff member verifies their eligibility. If all study eligibility criteria are satisfied, the study staff member opens the next envelope in the sequence and reveals the group assignment to the participant.

### Blinding

Full blinding is not possible for this trial. Study staff who interact closely with patients (e.g., by identifying and scheduling MBSR classes, or performing the randomization) are not blinded. Investigators, statisticians, programmers, and study staff who collect outcome measurements remain blinded to participant treatment assignment until the primary analyses are completed. If a serious adverse event occurs, the study physician (AA) can be unblinded as necessary.

### Study intervention

Participants in the active-treatment group attend a group MBSR class that follows the established protocol for the MBSR intervention as developed by Kabat-Zinn and colleagues at the Center for Mindfulness, University of Massachusetts Medical School [19]. These classes comprise weekly 2.5-h group sessions and a 1-day weekend retreat conducted by a trained MBSR teacher. As this study is designed as a pragmatic trial, participants attend a community-based class for which the instructor and the curricula have been vetted by the study MBSR expert (RS). There are numerous class options for participants within and outside the participating health systems and participants are free to choose any of the approved classes that best matches their availability. The cost of the MBSR classes is covered by study funds. A participant is considered to have successfully adhered to the intervention if they attend at least five of the eight weekly sessions and the 1-day weekend retreat (attendance records are provided by the MBSR instructors). The active treatment is in addition to usual care. The fidelity of the instructor to the vetted curricula was not monitored because of the large number of class options and the potential for such monitoring to become burdensome or intrusive.

Participants randomized to the control arm will continue with their usual activities and complete the study data-collection activities on a schedule identical to that of the MBSR-allocated participants. After the 8-month study period has passed, control-group participants are offered the opportunity to enroll in one of the approved community-based MBSR classes with costs covered by study funds (i.e., a wait-list control).

### Outcome measures

There are two sets of outcome measures for this pilot trial: those relevant to the feasibility objectives (recruitment and intervention adherence) and those relevant to the effectiveness of the MBSR intervention. Explicit criteria for judging the success of the proposed trial methods have been established a priori. Study methods will be deemed successful within each of the following attributes:

- Recruitment: Enrollment of at least 60 participants within any 36-week period or enrollment of at least 18 participants within any 9-week period.
- Intervention adherence: At least 80% of intervention-allocated participants completed at least five of the eight weekly MBSR classes and the 1-day weekend retreat.
- Primary effectiveness outcome: The primary outcome for assessing the clinical effectiveness of the MBSR intervention is the change in frequency of migraine episodes from baseline as measured by a 31-day headache diary at 4 months.
- Secondary effectiveness outcomes: Secondary measures of intervention effectiveness were selected to capture specific clinical and quality-of-life attributes that may be affected by the MBSR intervention. All secondary outcomes described below are collected and managed using Research Electronic Data Capture (REDCap) tools hosted at Sutter Health [20]. REDCap is a secure, web-based application designed to support data capture for research studies, providing (1) an intuitive interface for validated data entry, (2) audit trails for tracking data manipulation and export procedures, (3) automated export procedures for seamless data downloads to common statistical packages, and (4) procedures for importing data from external sources.
- Questionnaire Assessments: Questionnaires were chosen to harmonize with the National Institutes of Health (NIH) Patient-reported Outcomes Measurement Information System (PROMIS) measures [21] and the National Institute of Neurological Disorders and Stroke (NINDS) Common Data Elements for Headache Studies [22], supplemented with additional instruments to assess specific domains and for compatibility with prior migraine trials. All instruments are self-administered, and the total respondent burden was kept to less than 30 min. All instruments have undergone psychometric testing and have been found to be acceptable in terms of internal consistency, content validity, concurrent validity, and test–retest reliability. The instruments are summarized in Table 1.

**Table 1:** Outcome instruments for the Mindfulness and Migraine Study

Instrument	Domain	Number of items	Time (min)	Source (references)
Migraine Disability Assessment (MIDAS)	Headache	5	2	NINDS [22]
Headache Impact Test (HIT-6)	Headache	6	2	NINDS [22]
Visual analogue scale	Pain	1	1	NINDS [22]
Medical Outcomes Study Short Form 12 (SF-12)	Function	12	4	NINDS [22]
Work Productivity and Activity Impairment (WPAI)	Productivity	6	2	[23]
Migraine-Specific Quality of Life (MSQ)	Quality of life	14	4	NINDS [22]
PROMIS Depression Short Form	Depression	8	3	PROMIS [21]
PROMIS Anxiety Short Form	Anxiety	8	3	PROMIS [21]
Perceived Stress Scale (PSS-10)	Stress	10	3	[25, 26]
Mindful Attention Awareness Scale (MAAS)	Mindfulness	15	5	[27, 28]
Total	–	85	29	–

*NINDS* National Institute of Neurological Disorders and Stroke, *PROMIS* Patient-reported Outcomes Measurement Information System

### Notes Regarding the non-NINDS/non-PROMIS measures

Reduced work productivity is a major consequence of poorly treated migraine and an outcome that may be amenable to a mind–body intervention such as MBSR. To measure this construct, we use the well-validated Work Productivity and Activity Impairment instrument (WPAI), which is widely used in the work productivity literature [23] and has been advocated specifically for use in migraine studies [24]. The Perceived Stress Scale (PSS-10) is the most widely used instrument for assessing perceptions of stress and has been used successfully in prior studies of meditation [25, 26]. The Mindful Attention Awareness Scale (MAAS) was developed as a global measure of mindfulness and has been successfully validated for use in clinical studies [27, 28].

#### Medication use

Data are collected on the use of analgesics and migraine-specific preventive and rescue medications using the 31-day headache diaries at all visits. There are no study medication restrictions; the protocol does not attempt to influence medications prescribed by the participants’ physicians. Medication use by all participants is tracked with questionnaires and by extracting all dispensed prescription medications from the EHR of both health systems.

#### Actigraph

Each participant is asked to wear an actigraph (GENEActiv™ accelerometer; Activinsights, Kimbolton, Cambridgeshire, U.K.) on their wrist for 2 weeks at baseline and for 2 weeks at the 3-month visit. The actigraphy data (an exploratory outcome) is collected for objective assessments of sleep quality in both groups.

#### Closeout interviews

We will conduct a single focus group with a random sample of participants who completed all phases of the study to gain a better understanding of the qualitative aspects of the trial and to learn about participants’ experience with their MBSR classes and which aspects of the trial were particularly successful and which could be improved. In addition, we will individually interview up to 10 participants who did not complete the study to gain a better understanding of personal barriers to successful completion.

### Clinic procedures and assessment schedule

**Fig. 2:**
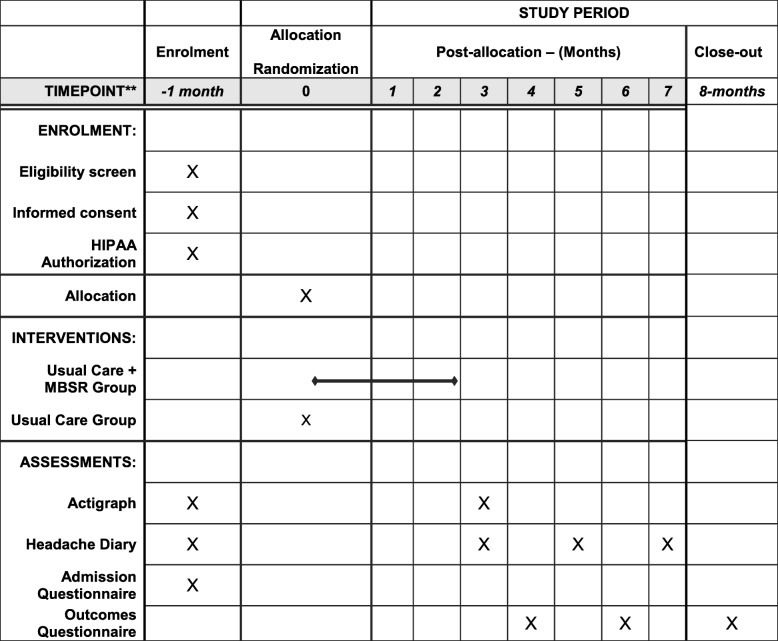
SPIRIT figure. HIPAA Health Insurance Portability and Accountability Act, MBSR Mindfulness-based stress reduction

This trial includes a telephone screening call, an eligibility screening visit, a 1-month (31-day) run-in period, a randomization visit, then an 8-month follow-up period that includes four in-person visits. The content of each assessment is described below, and the timing described in the Spirit figure (Additional file 1; Fig. 2). The workflow of randomized participants is illustrated in Fig. 1.

#### Telephone screen

All potentially eligible patients who are interested in the study are screened by telephone interview. The telephone screen informs potential participants about the trial, answers any potential questions, and determines whether participants meet telephone-assessable inclusion and exclusion criteria. Study staff track and record information in a recruitment log on every telephone screen conducted. If the potential participant meets the preliminary eligibility criteria based on the telephone screen, they may continue the study eligibility process with an in-person first screening visit.

#### Initial assessment

The initial assessment takes place over a 31-day run-in period. This multi-component procedure evaluates a potential participant’s study commitment and confirms their eligibility. This process includes a first screening visit and an enrollment and randomization visit.

#### First screening visit

The first screening visit is scheduled during the telephone screen and takes approximately 1 hour. At this visit, all eligibility criteria are reviewed and validated, and the potential participant’s commitment and availability are assessed. Potential participants who meet the initial study criteria are given a copy of the informed consent form, and the study staff reviews the document with them. If they agree to and sign the consent form, they are invited to begin the run-in period. This screening visit is conducted by a study coordinator who is trained in both clinical trials and in MBSR.

#### Run-in period

Consented participants begin the 31-day run-in period prior to randomization. Patients in this phase complete a daily headache diary containing five questions related to headache frequency and severity, and medication use. Participants are also given an actigraph to wear for the first 14 days of the run-in period.

#### Enrollment and randomization visit

Participants who record between 4 and 14 headache days during the run-in period are eligible to proceed to randomization. Study participants are randomized into one of two study arms: (1) the MBSR intervention plus usual care or (2) usual care alone. Participants complete questionnaires regarding their health and headaches, which take less than 30 min to complete.

#### Intervention period

Participants randomized to the MBSR arm are registered to attend a public MBSR class. In most cases, there is a waiting period of up to 3 months before the class begins. The 8-month follow-up period begins on the official first MBSR class date. Participants have the option to conduct the intervention-period study visits virtually.

#### Follow-up visits

Three months after the start of the MBSR classes, participants attend for a follow-up visit and are asked to wear the actigraph again for 14 days. We collect any feedback on the MBSR class for those in the intervention arm. The second headache diary (for measurement of the primary study outcome) is provided.

Four months after the start of the MBSR classes, participants attend for a follow-up visit. They return their actigraph and the second headache diary, and complete the online questionnaires. They are also asked about any adverse events.

Six months after the start of the MBSR classes, participants attend for a follow-up visit. They return their third headache diary and complete the online questionnaires. They are asked about any adverse events.

Eight months after the start of the MBSR classes, participants attend for a follow-up visit. They return their fourth and final headache diary and complete the online questionnaires. They are asked about any adverse events. Participants in the control group are offered the MBSR course, and all participants are asked if they would be willing to take part in a focus group.

#### Retention and adherence to MBSR

For each follow-up visit, telephone calls are made to arrange a visit time. The study research assistant will make phone calls until the participant is reached and an appointment is scheduled. After the follow-up window is closed, if the participant has not been contacted, or they have asked to be dropped from the study, the participant is marked as a withdrawal.

The teachers of the individual MBSR classes are asked to handle absenteeism as usual, and provide attendance records for their students in this study.

#### Compensation

Participants are compensated $25 for completing the 4-month data collection, $50 for the 6-month data collection and $100 for the 8-month closeout data collection. Among those individuals who complete all aspects of the study, we will randomly select 15 participants to take part in a focus group for which an additional $100 will be given.

### Statistical procedures

#### Sample size

The sample size of approximately 60 participants for this pilot study was determined in collaboration with the funding agency and was felt to be large enough to gauge whether the community-based classes would be feasible for a full-scale clinical trial intervention. This sample will allow us to estimate an adherence rate of 0.8 with confidence limits ±0.1 around the point estimate.

#### Statistical analysis

As a pilot trial, the feasibility objectives are central and are focused on recruitment, retention, intervention adherence, and data collection. Recruitment rates are calculated as the mean number of participants enrolled into the trial per week divided by the mean number of patients screened per week. In addition, we examine success in meeting the stated recruitment goals described above. Adherence to the intervention is measured dichotomously (as the proportion satisfying the noted adherence definition) and as the distribution of the proportion of all classes completed. Data-collection success rates are estimated by calculating the proportion of all forms fully completed by participants who remain in the trial (i.e., who have not withdrawn), and the proportion of all forms that should have been completed if retention and data collection were 100%. No formal tests of intervention effectiveness are planned, given the intentionally underpowered pilot nature of the study [29, 30].

### Data and safety issues

#### Adverse events

At the 4-, 6-, and 8-month visits, participants are asked about any untoward medical events that have occurred since the prior visit, as well as emergency department visits and hospitalizations. Details of all adverse events are recorded and the study physician (AA) will assess each event for seriousness, severity, attribution, and preexisting status, and whether the participant’s continued activities in the trial should be modified or terminated. All adverse events are followed until resolution or the end of the participant’s follow-up period. All serious adverse events and unanticipated problems are reported according to IRB mandates, funding institute directives, and data and safety monitoring board (DSMB) requirements. Non-serious adverse events are reported to the local IRB annually and at all formal meetings of the DSMB.

#### Data and safety

A DSMB is constituted for this pilot trial, consisting of one experienced clinical trialist, a migraine clinician/content expert, and a statistician. Both blinded and unblinded data are provided, per the determination of the DSMB. The meeting schedule and content of the DSMB reports are consistent with the guidelines of the funding institute and will be modified and approved as necessary by the DSMB itself. The content of the DSMB reports consists of all relevant trial progress information, safety data, and measures of data completeness and quality.

## Discussion

Migraine headaches are one of the most common neurological conditions seen in primary and specialty neurological care. They are often distressing. Despite the availability of several effective preventive and rescue medications, many patients continue to suffer with migraine symptoms as these therapeutics are insufficiently effective for many patients, are often associated with bothersome side effects, and can be very expensive. In addition, many patients prefer interventions that do not involve medications and over which they have control [31].

MBSR has been shown to provide clinically meaningful benefits with few adverse effects for patients with a wide variety of chronic-pain syndromes such as chronic low back pain [32–34] and fibromyalgia [35, 36], among other conditions. To date, however, there are no results from large-scale fully powered clinical trials of an MBSR intervention for patients with frequent migraine headaches. Research into the clinical and biological effects of stress-altering interventions is in the early stages. One small pilot trial (*N* = 19) found an indication of potential clinical benefits of MBSR [11]. Two additional trials of MBSR among migraine patients are also in progress, including one trial with clinical outcomes and another on cross-sectional imaging outcomes (https://clinicaltrials.gov/ct2/show/record/NCT02695498 and https://clinicaltrials.gov/ct2/show/record/NCT02133209).

Such a trial would require substantial resources and patient commitment. Prior to embarking on such an endeavor, it is prudent to ensure that study methods are robust and will likely lead to a successful, valid phase III clinical trial.

This protocol describes a pilot trial to compare the effect of community-based MBSR classes with usual care to usual care alone as a preventive treatment for patients with moderately severe migraine headaches. The trial is designed to be highly pragmatic, recruiting a community-based sample of participants with few restrictions on adjunctive therapies and using community-based MBSR classes that represent the typical experience of patients who might choose to practice MBSR for migraine prevention. Thus, the patient population and the intervention are likely to be representative of the usual clinical environment in which such an intervention would be offered. Generalizability is also enhanced by the inclusion of multiple independent recruitment strategies, allowing for comparison of alternative recruitment approaches. The study includes a wide range of clinically relevant outcomes and includes a test of the feasibility of digital-sensor technology (actigraphs) to give a better understanding of the physiological responses to an MBSR intervention.

Note that there are several limitations to our approach for this pilot study. First, because we do not have an active comparison arm or attention control, the participants cannot be blinded to their treatment. There may be non-specific or placebo effects associated with the treatment, which we do not want to lose in an analysis of the effectiveness data in a fully powered trial [37]. We did not include measures of expectancy. These should be included in a fully powered trial since they may shed light on observed responses. We accept a clinical diagnosis of migraine for eligibility and did not include a baseline validation by a neurologist or headache specialist. We have also discovered that the time between randomization and intervention-group participants starting their MBSR classes was longer than expected (often several weeks), introducing an imbalance and potential bias from differential follow-up periods. This problem will require a design change in a fully powered study (such as matching follow-up periods for intervention and control participants). We also did not collect detailed data on all potential co-interventions in the control group nor the characteristics of the classes in the intervention group. Finally, we did not include any interventions to promote adherence to the home diary collection. As adherence was a feasibility outcome for this study, if we learn that adherence to completing the diaries is suboptimal, we will include reminders in the fully powered trial.

This pilot trial was designed to replicate, on a limited scale, all of the essential elements of a fully powered effectiveness study of MBSR for patients with migraine, including recruitment, run-in, randomization, application of the intervention, data collection (including all patient-reported outcomes and mechanistic studies), adherence monitoring, retention, study closeout, and regulatory compliance (including a fully constituted DSMB). Lessons learned in this pilot study will be incorporated into the design of future phase III studies. The results of this pilot study will inform future studies of MBSR for migraine and other related conditions, helping ensure that future studies of this promising intervention are valid and generalizable to usual clinical settings.

### Trial status

This trial is registered at clinicaltrials.gov (NCT02824250; https://clinicaltrials.gov/ct2/show/NCT02824250), and was first posted on 6 July 2016. The first participant gave their consent on 2 February 2017 and was randomized on 9 March 2017. Recruitment was completed 21 September 2018. The most recent version of the protocol (V2.0) was approved by the Sutter Health IRB on 9 March 2018 (Sutter IRB #: 2016.070EXP IRBNet #: 897613).

## Additional file

Additional file 1: SPIRIT checklist. (DOC 123 kb)

## Abbreviations

DSMB: Data and safety monitoring board
EHR: Electronic health records
HIT: Headache Impact Test
IRB: Institutional review board
M&M: Mindfulness and Migraine
MAAS: Mindful Attention Awareness Scale
MBSR: Mindfulness-based stress reduction
MIDAS: Migraine Disability Assessment
MSQ: Migraine-specific quality of life
NIH: National Institutes of Health
NINDS: National Institute of Neurological Disorders and Stroke
PROMIS: Patient-reported Outcomes Measurement Information System
PSS-10: Perceived Stress Scale
REDCap: Research electronic data capture
SF-12: Medical Outcomes Study Short Form 12
WPAI: Work Productivity and Activity Impairment

## References

[1] Lipton RB, Bigal ME, Diamond M, Freitag F, Reed ML, Stewart WF (2007). Migraine prevalence, disease burden, and the need for preventive therapy. Neurology..

[2] Goldberg LD (2005). The cost of migraine and its treatment. Am J Manag Care.

[3] Stovner L, Hagen K, Jensen R, Katsarava Z, Lipton R, Scher A (2007). The global burden of headache: a documentation of headache prevalence and disability worldwide. Cephalalgia..

[4] Merikangas KR (2013). Contributions of Epidemiology to Our Understanding of Migraine. Headache.

[5] Burch R, Rayhill M (2018). New preventive treatments for migraine. BMJ.

[6] Theeler BJ, Kenney K, Prokhorenko OA, Fideli US, Campbell W, Erickson JC (2010). Headache triggers in the U.S. military. Headache..

[7] Sauro KM, Becker WJ (2009). The stress and migraine interaction. Headache..

[8] Greeson J, Eisenlohr-Moul T (2014). Mindfulness-Based Stress Reduction for Chronic Pain. Mindfulness-Based Treatment Approaches: Clinician’s Guide to Evidence Base and Applications.

[9] Gotnick R, Chu P, Busschbach J, Benson H, Fricchione G, Standardised HM. Mindfulness-Based Interventions in Healthcare: An Overview of Systematic Reviews and Meta-Analyses of RCTs. PLoS One. 2015;10(4):1–17. 10.1371/journal.pone.0124344 PMC4400080 25881019

[10] Rosenzweig S, Greeson JM, Reibel DK, Green JS, Jasser SA, Beasley D (2010). Mindfulness-based stress reduction for chronic pain conditions: variation in treatment outcomes and role of home meditation practice. J Psychomatic Res.

[11] Wells RE, Burch R, Paulsen RH, Wayne PM, Houle TT, Loder E (2014). Meditation for migraines: a pilot randomized controlled trial. Headache..

[12] Lauche R, Cramer H, Paul A, Dobos GJ, Rampp T (2012). Introducing integrative integrated migraine care (IIMC): a model and case presentation. Eur J Integr Med.

[13] Oberg EB, Rempe M, Bradley R (2013). Self-directed mindfulness training and improvement in blood pressure, migraine frequency, and quality of life. Glob Adv Health Med.

[14] Schmidt S., Simshäuser K., Aickin M., Lüking M., Schultz C., Kaube H. (2010). Mindfulness-based stress reduction is an effective intervention for patients suffering from migraine—Results from a controlled trial. European Journal of Integrative Medicine.

[15] Eldridge SM, Lancaster GA, Campbell MJ, Thabane L, Hopewell S, Coleman CL (2016). Defining Feasibility and Pilot Studies in Preparation for Randomised Controlled Trials: Development of a Conceptual Framework. PLOS ONE.

[16] Schulz KF, Altman DG, Moher D (2010). CONSORT 2010 Statement: updated guidelines for reporting parallel group randomised trials. BMJ..

[17] Pressman A, Jacobson A, Eguilos R, Gelfand A, Huynh C, Hamilton L (2016). Prevalence of migraine in a diverse community-electronic methods for migraine ascertainment in a large integrated health plan. Cephalalgia..

[18] StataCorp (2015). Stata Statistical Software: Release 14.

[19] History of MBSR [Internet]. University of Massachusetts Medical School. 2016 [cited 2018 Sep 6]. Available from: https://www.umassmed.edu/cfm/mindfulness-based-programs/mbsr-courses/about-mbsr/history-of-mbsr/

[20] Harris PA, Taylor R, Thielke R, Payne J, Gonzalez N, Conde JG (2009). Research electronic data capture (REDCap)--a metadata-driven methodology and workflow process for providing translational research informatics support. J Biomed Inform.

[21] Weinfurt, Cella. PROMIS Dynamic Tools to MEasure Health Outcomes from the Patient Perspective [Internet]. NIH; [cited 2015 Jun 1]. Available from: http://www.nihpromis.org

[22] Odenkirchen J. NINDS Common Data Elements. Harmonizing Information. Streamlining Research. [Internet]. NIH; 2015 [cited 2015 Jun 1]. Available from: http://www.commondataelements.ninds.nih.gov/ProjReview.aspx#tab=Introduction

[23] Reilly MC, Zbrozek AS, Dukes EM (1993). The validity and reproducibility of a work productivity and activity impairment instrument. Pharmacoeconomics..

[24] Loeppke R, Hymel PA, Lofland JH, Pizzi LT, Konicki DL, Anstadt GW (2003). Health-related workplace productivity measurement: general and migraine-specific recommendations from the ACOEM Expert Panel. J Occup Environ Med.

[25] Lane JD, Seskevich JE, Pieper CF (2007). Brief meditation training can improve perceived stress and negative mood. Altern Ther Health Med.

[26] Marcus MT, Fine PM, Moeller FG, Khan MM, Pitts K, Swank PR (2003). Change in Stress Levels Following Mindfulness-based Stress Reduction in a Therapeutic Community. Addict Disord Treat.

[27] Brown KW, Ryan RM (2003). The benefits of being present: Mindfulness and its role in psychological well-being. J Pers Soc Psychol.

[28] Carlson LE, Brown KW (2005). Validation of the Mindful Attention Awareness Scale in a cancer population. J Psychosom Res.

[29] Kraemer HC, Mintz J, Noda A, Tinklenberg J, Yesavage JA (2006). Caution regarding the use of pilot studies to guide power calculations for study proposals. Arch Gen Psychiatry.

[30] Leon AC, Davis LL, Kraemer HC (2011). The role and interpretation of pilot studies in clinical research. J Psychiatr Res.

[31] Cowan RP (2014). CAM in the Real World: You May Practice Evidence-Based Medicine, But Your Patients Don’t. Headache.

[32] Morone NE, Greco CM, Moore CG, Rollman BL, Lane B, Morrow LA (2016). A Mind-Body Program for Older Adults With Chronic Low Back Pain: A Randomized Clinical Trial. JAMA Intern Med.

[33] Zgierska AE, Burzinski CA, Cox J, Kloke J, Stegner A, Cook DB (2016). Mindfulness Meditation and Cognitive Behavioral Therapy Intervention Reduces Pain Severity and Sensitivity in Opioid-Treated Chronic Low Back Pain: Pilot Findings from a Randomized Controlled Trial. Pain Med.

[34] Cherkin DC, Sherman KJ, Balderson BH, Cook AJ, Anderson ML, Hawkes RJ (2016). Effect of Mindfulness-Based Stress Reduction vs Cognitive Behavioral Therapy or Usual Care on Back Pain and Functional Limitations in Adults With Chronic Low Back Pain: A Randomized Clinical Trial. JAMA..

[35] Van Gordon W, Shonin E, Dunn TJ, Garcia-Campayo J, Griffiths MD (2017). Meditation awareness training for the treatment of fibromyalgia syndrome: A randomized controlled trial. Br J Health Psychol.

[36] Cash E, Salmon P, Weissbecker I, Rebholz WN, Bayley-Veloso R, Zimmaro LA (2015). Mindfulness meditation alleviates fibromyalgia symptoms in women: results of a randomized clinical trial. Ann Behav Med.

[37] Avins AL, Cherkin DC, Sherman KJ, Goldberg H, Pressman A. Should we reconsider the routine use of placebo controls in clinical research? Trials. 2012;13(1) Available from: http://trialsjournal.biomedcentral.com/articles/10.1186/1745-6215-13-44. [cited 2019 Jan 31]. 10.1186/1745-6215-13-44 PMC3404895 22540350

